# Obesity influencing circulating levels of nutrients: Evidence from Mendelian randomization study

**DOI:** 10.1097/MD.0000000000039594

**Published:** 2024-09-13

**Authors:** Guie Gao, Ruzhen Ou, Wenhui Chen

**Affiliations:** a Department of Operating Room, The First Affiliated Hospital of Jinan University, Guangzhou, China; b Department of Nursing, The First Affiliated Hospital of Jinan University, Guangzhou, China; c Department of Metabolic and Bariatric Surgery, The First Affiliated Hospital of Jinan University, Guangzhou, China.

**Keywords:** causality, genes, Mendelian randomization, nutrients, obesity

## Abstract

Observational studies have established that obesity is associated with nutritional deficiencies, but the exact causality remains uncertain. Thus, this Mendelian randomization (MR) study aimed to identify the causal associations between obesity and circulating levels of nutrients. Single-nucleotide polymorphisms associated with obesity (body mass index and waist-hip ratio), were extracted from a genome-wide association study of 694,649 European ancestry. Summary-level data for minerals (copper, selenium, zinc, calcium, magnesium, and potassium), and vitamins (folate, vitamins A, C, E, B6, and B12), albumin were obtained from the publicly available integrative epidemiology unit OpenGWAS database psychiatric genomics consortium. Inverse-variance weighted method several sensitivity analyses were conducted. Genetically predicted higher body mass index significantly decreased circulating levels of magnesium (*β* = −0.07, 95% confidence interval [CI]: −0.10 to −0.03, *P* = 1.47 × 10^−4^), folate (*β* = −0.07, 95% CI: −0.10 to −0.04, *P* = 5.61 × 10^−5^), vitamin A (*β* = −0.11, 95% CI: −0.14 to −0.07, *P* = 3.10 × 10^−9^), vitamin E (*β* = −0.10, 95% CI: −0.13 to −0.06, *P* = 1.84 × 10^−8^), albumin (*β* = −0.15, 95% CI: −0.17 to −0.12, *P* = 9.89 × 10^−28^); whereas genetically predicted higher waist-hip ratio decreased circulating levels of magnesium (*β* = −0.07, 95% CI: −0.11 to −0.02, *P* = 1.87 × 10^−3^), folate (*β* = −0.07, 95% CI: −0.11 to −0.03, *P* = 9.87 × 10^−4^), vitamin C (*β* = −0.08, 95% CI: −0.12 to −0.04, *P* = 2.40 × 10^−4^), albumin (*β* = −0.08, 95% CI: −0.11 to −0.04, *P* = 3.72 × 10^−5^). The study supports a causal effect of obesity on lower circulating levels of nutrients. Our findings highlight the necessity of adjuvant nutrients in obesity management.

## 1. Introduction

Obesity is a chronic disease with excessive fat accumulation in the body. As a condition with energy excess, obesity seems unrelated to nutritional deficiencies. Paradoxically, in the real world, many patients with obesity are observed to be presented with nutritional deficiencies. A large number of observational studies have reported deficiencies were found in iron, folate, vitamin D, and vitamin B12 in patients with obesity.^[1–5]^ It is estimated that anemia affects about 10% to 15% of bariatric patients.^[6]^ A systematic review including 30 studies demonstrated that vitamin D deficiency ranged from 13% to 90% in patients with obesity before bariatric surgery.^[7]^ The deficits in nutrients will lead to a range of adverse clinical conditions. For example, the deficits of iron, folate, and vitamin B12 can cause anemia-related symptoms, including fatigue, dizziness, poor memory, and exertional dyspnea.^[8]^ Vitamin D deficiency can cause systemic disorders of calcium and phosphorus metabolism, leading to osteoporosis and bone resorption disorders.^[9]^ The precise reasons for nutritional deficiencies in patients with obesity remain unclear. The consumption of high calorie but poor essential nutrients contributed to nutritional deficiencies in patients with obesity.^[10]^ Additionally, some behavioral factors may explain these findings, including picky eaters, low sunlight exposure, and outdoor activity.^[11]^ However, the exact causality cannot be further clarified. In clinical practice, further evidence is required to determine whether routine nutrient supplementation is necessary for overweight or obese individuals.

Mendelian randomization (MR) is a novel method to determine the causal effect of exposure on the outcome by using single-nucleotide polymorphisms (SNPs) as genetic instruments. Since leveraging random allocation of alleles during meiosis and fertilization, MR studies could solve bias from reverse causation and unobserved confounders in observational epidemiology.^[12]^ So far, some previous MR studies had indicated that genetically predicted obesity was causally associated with lower levels of iron and vitamin D.^[13,14]^ Still, the causal relationship of obesity with circulating levels of nutrients has not been systematically evaluated. Therefore, in this study, we aimed to explore the causal relationship between genetically predicted obesity traits and the genetically predicted circulating levels of nutrients by conducting a comprehensive 2-sample MR analysis.

## 2. Materials and methods

### 2.1. Study design

**Figure 1.**
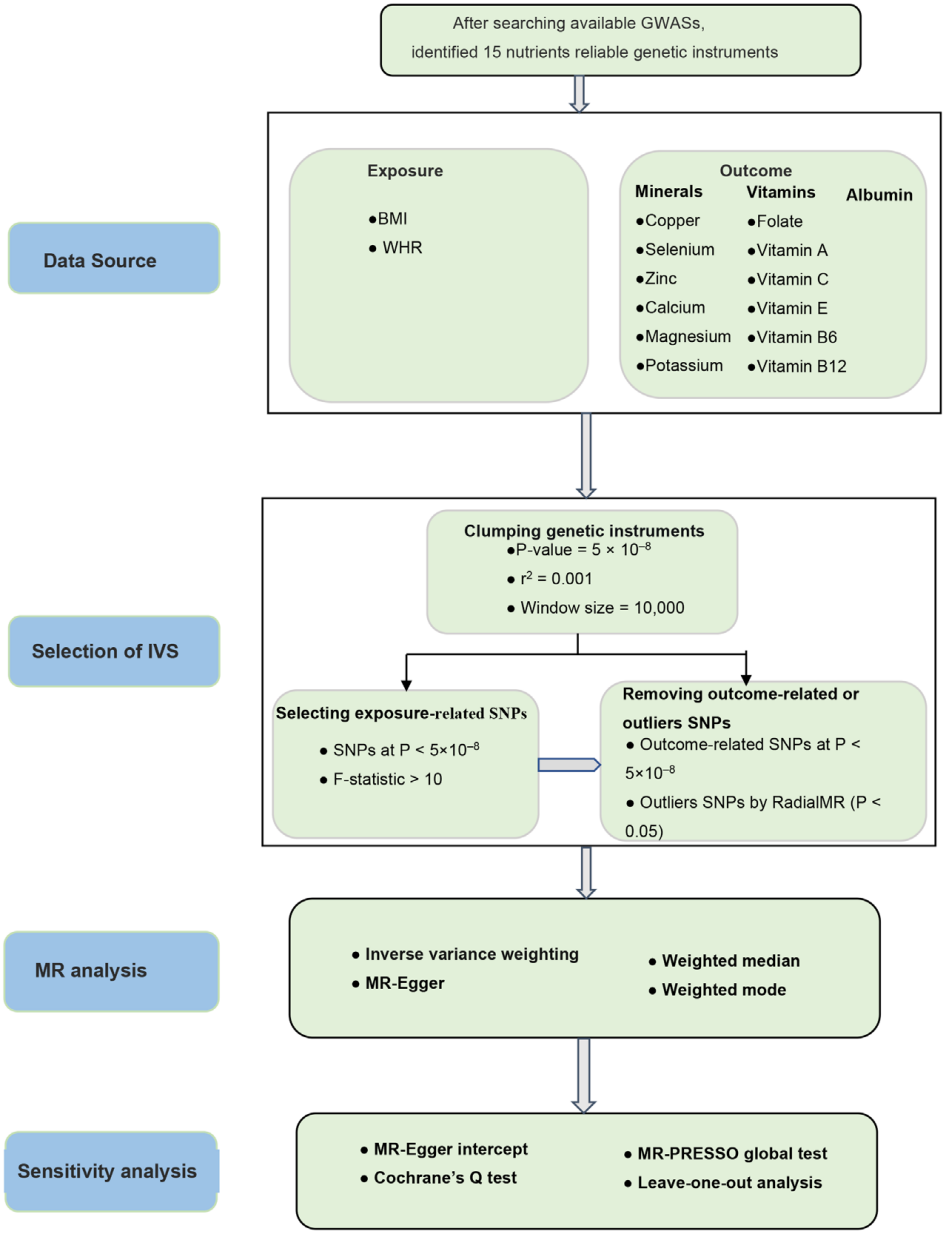
The study framework chart. BMI = body mass index, WHR = waist-to-hip ratio.

The current MR study is reported according to Strengthening the Reporting of Observational Studies in Epidemiology.^[15]^ MR analysis is based on 3 basic assumptions: genetic variants strongly associated with the exposure; genetic variants are independent from confounding factors; genetic variants influence outcomes only through the selected exposure. All data utilized in this work are from publicly available among European populations, for which additional ethical approval and informed consent were obtained in original studies. The overall design of our study is illustrated in Figure 1.

### 2.2. Data source and instrumental variable selection

We obtained the genetic instruments for obesity traits from the Genetic Investigation of Anthropometric Traits Consortium, a recent meta-analysis including 694,649 individuals of European ancestry,^[16]^ which were available at online database (https://portals.broadinstitute.org/collaboration/giant/index.php/GIANT_consortium_data_files). Overall obesity was measured by body mass index (BMI) which calculated by dividing weight [kg] by squared height [m^2^]; while abdominal obesity was measured by waist-hip ratio (WHR) which calculated by dividing waist circumference [cm] by hip circumference [cm].

We searched for published literature on nutritional deficiencies in patients with obesity,^[17,18]^ and initially retrieved the available summary-level genome-wide association study (GWAS) summary statistics for 17 nutrients, including minerals (copper, selenium, zinc, calcium, magnesium, iron, potassium); vitamin (folate, vitamin A, vitamin C, vitamin D, vitamin E, vitamin B6, vitamin B12); albumin and ferritin. We excluded iron, ferritin, and vitamin D from MR analysis because the previous MR studies have already explored the role of obesity on circulating levels of iron, ferritin, and vitamin D.^[13,14,19,20]^ The summary data were obtained from the Integrative Epidemiology Unit OpenGWAS database (https://gwas.mrcieu.ac.uk/, accessed on April 10, 2023), the GWAS Catalog and PubMed. The OpenGWAS database has collected 245,745,128,198 genetic associations from 42,346 GWAS summary datasets, for query or download. The datasets utilized in our study are presented in Table 1.

**Table 1:** Characteristics of datasets used in our study.

Traits	GWAS ID	Year	Consortium	Population	Sample size
Exposure					
BMI	NA	2019	GIANT	European	806,834
WHR	NA	2019	GIANT	European	697,734
Outcome					
Copper	ieu-a-1073	2013	NA	European	2603
Selenium	ieu-a-1077	2013	NA	European	2603
Zinc	ieu-a-1079	2013	NA	European	2603
Calcium	ukb-b-8951	2018	MRC-IEU	European	64,979
Magnesium	ukb-b-7372	2018	MRC-IEU	European	64,979
Potassium	ukb-b-17881	2018	MRC-IEU	European	64,979
Folate	ukb-b-11349	2018	MRC-IEU	European	64,979
Vitamin A	ukb-b-17406	2018	MRC-IEU	European	64,979
Vitamin C	ukb-b-19390	2018	MRC-IEU	European	64,979
Vitamin E	ukb-b-6888	2018	MRC-IEU	European	64,979
Vitamin B 6	ukb-b-7864	2018	MRC-IEU	European	64,979
Vitamin B12	ukb-b-19524	2018	MRC-IEU	European	64,979
Albumin	met-d-Albumin	2020	NA	European	115,060

BMI = body mass index, GIANT = Genetic Investigation of Anthropometric Traits, IEU = integrative epidemiology unit, MRC = Medical Research Council, NA = not available, WHR = waist-to-hip ratio.

We extracted genetic instruments with genome-wide significance (*P* < 5 × 10^−8^) for each exposure and removed SNPs with linkage disequilibrium (*r*^2^ > 0.001 and clumping window <10,000 kb). In addition, we calculated the *F*-statistics using the formula *F *= *β^2^*/*σ^2^* (*β*: beta for SNP-exposure effect estimate, *σ*: standard deviation for variant) to assess the strength of genetic instruments, and excluded SNPs with *F* <10 to avoid the bias of weak instruments. When harmonizing exposure and outcome data, palindromic SNPs with intermediate allele frequencies and outcome-related SNPs (*P < *5 × 10^−8^) were further removed. To enhance the robustness of MR analysis, we conducted heterogeneity test (modified *Q* statistics) by using RadialMR analysis and removed outliers with a *P* < .05. Finally, the remaining SNPs were performed for MR analysis.

### 2.3. Statistical analysis

The primary MR analyses were performed by using the inverse-variance weighted method to explore the causal relationships of genetically predicted obesity with circulating levels of nutrients. This method may be biased because it assumes that all SNPs are valid instruments and no pleiotropy exists.^[21]^ Thus, we additionally performed several complementary methods, including MR-Egger regression,^[22]^ weighted median,^[23]^ and weighted mode.^[24]^ The MR-Egger regression also can provide precise estimates despite the presence of horizontal pleiotropy. The weighted median method can provide consistent estimates if over half weight comes from valid SNPs. The weighted mode method can estimate the causal effect even though most of the SNPs are invalid.

We further conducted a series of sensitivity analyses to evaluate the robustness of the MR results. First, The MR-Pleiotropy Residual Sum and Outlier methods (MR-PRESSO) were applied to identify horizontal pleiotropic outliers of SNPs, and obtain the corrected results in the absence of outliers.^[25]^ Second, the intercept test of MR-Egger regression was used to detect possible directional pleiotropy.^[22]^ Third, heterogeneity among estimates was assessed with Cochran *Q* value, with *P* < .05 indicating the presence of heterogeneity. Finally, the leave-one-out analysis was conducted to evaluate the influence of the single SNP on the observed results.

We also performed post hoc power analysis using an online power calculation tool (https://sb452.shinyapps.io/power/) (Table S5, Supplemental Digital Content, http://links.lww.com/MD/N578, which illustrates statistical power calculations for the results of obesity on circulating levels of nutrients). An observed *P* value of <.05 was considered nominally significant, whereas statistical significance corrected for multiple testing was set at *P* value <1.92 × 10^−3^ (.05/26). All MR analyses were performed using “TwoSampleMR,” “MR-PRESSO,” and “RadialMR” packages in R software (version 4.2.0).

## 3. Results

After performing clumping and linkage disequilibrium, a total of 544 SNPs of BMI and 350 SNPs of WHR were identified, and the explained variances were 4.72% to 2.95% (Tables S1–S2, Supplemental Digital Content, http://links.lww.com/MD/N578, which illustrates genetically predicted BMI and WHR). All the *F*-statistics for these SNPs were >10, suggesting strong instruments.

### 3.1. The causal effect of BMI on circulating levels of nutrients

**Figure 2.**
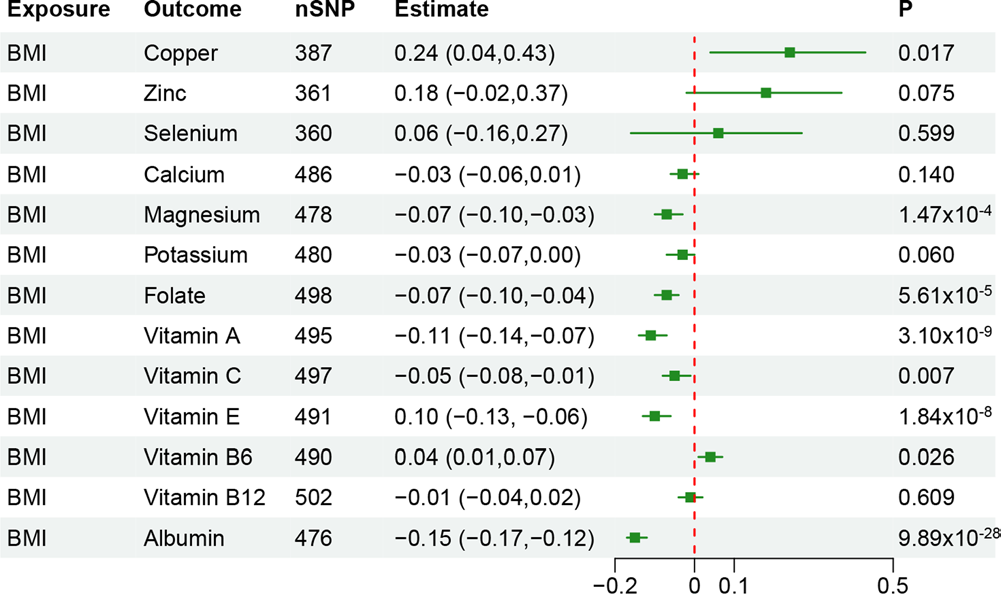
Mendelian randomization estimates for the associations of BMI on circulating levels of nutrients. BMI = body mass index.

After correction for multiple testing in the main MR analysis, we found genetic predicted higher BMI was significantly associated with lower circulating levels of magnesium (*β* = −0.07, 95% confidence interval [CI]: −0.10 to −0.03, *P* = 1.47 × 10^−4^), folate (*β* = −0.07, 95% CI: −0.10 to −0.04, *P* = 5.61 × 10^−5^), vitamin A (*β* = −0.11, 95% CI: −0.14 to −0.07, *P* = 3.10 × 10^−9^), vitamin E (*β* = −0.10, 95% CI: −0.13 to −0.06, *P* = 1.84 × 10^−8^), albumin (*β* = −0.15, 95% CI: −0.17 to −0.12, *P* = 9.89 × 10^−28^). Additionally, we also observed that predicted higher BMI was a nominally significant association with circulating levels of copper, vitamin C, and vitamin B6 (Fig. 2). See Table S3, Supplemental Digital Content, http://links.lww.com/MD/N578, which illustrates MR analysis results of obesity on circulating levels of nutrients, and Figure S1, Supplemental Digital Content, http://links.lww.com/MD/N577, which illustrates scatter plots of significant and nominal significant estimates about obesity on circulating levels of nutrients.

In sensitivity analyses, no heterogeneity was detected in all main MR analysis (*P* >.05). The MR-Egger intercept test suggested there was the potential directional pleiotropy in significant MR results for magnesium and vitamin E, whereas no outliers were observed in the MR-PRESSO analysis (*P* > .05). (See Table S4, Supplemental Digital Content, http://links.lww.com/MD/N578, which illustrates pleiotropy assessment for the results of obesity on circulating levels of nutrients.) The leave-one-out analyses demonstrated that the removal of SNP did not substantially influence the results. (See Figure S2, Supplemental Digital Content, http://links.lww.com/MD/N577, which illustrates leave-one-out plots of significant and nominal significant estimates about obesity on circulating levels of nutrients.)

### 3.2. The causal effect of WHR on circulating levels of nutrients

**Figure 3.**
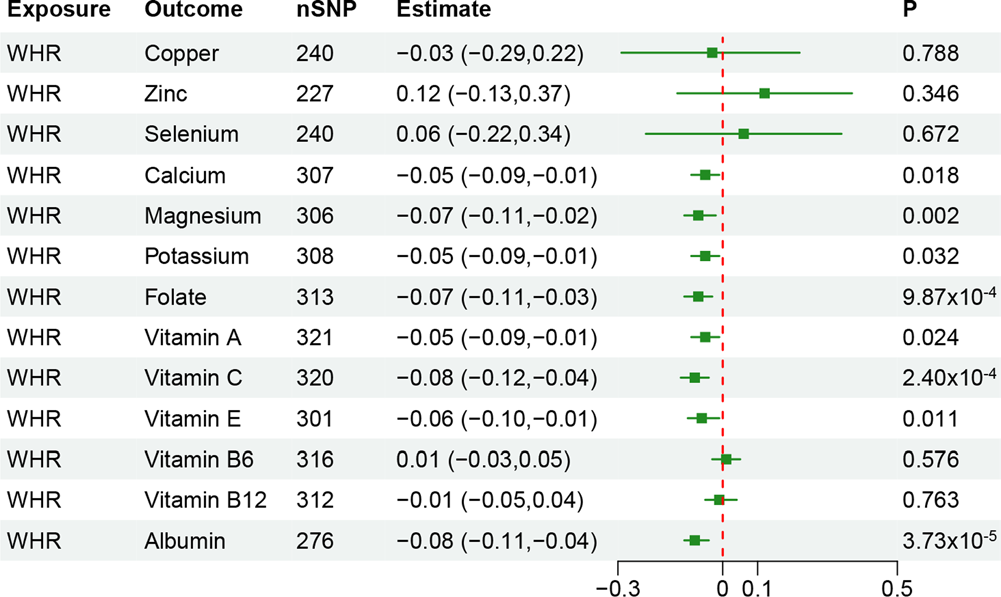
Mendelian randomization estimates for the associations of WHR on circulating levels of nutrients. WHR = waist-to-hip ratio.

Genetic predicted higher WHR was significantly associated with lower circulating levels of magnesium (*β* = −0.07, 95% CI: −0.11 to −0.02, *P* = 1.87 × 10^−3^), folate (*β* = −0.07, 95% CI: −0.11 to −0.03, *P* = 9.87 × 10^−4^), vitamin C (*β* = −0.08, 95% CI: −0.12 to −0.04, *P* = 2.40 × 10^−4^), albumin (*β* = −0.08, 95% CI: −0.11 to −0.04, *P* = 3.72 × 10^−5^). Genetic predicted higher WHR were nominally significant association with circulating levels of calcium, potassium, vitamin A, and vitamin E (Fig. 3). See Table S3, Supplemental Digital Content, http://links.lww.com/MD/N578, which illustrates MR analysis results of obesity on circulating levels of nutrients, and Figure S1, Supplemental Digital Content, http://links.lww.com/MD/N577, which illustrates scatter plots of significant and nominal significant estimates about obesity on circulating levels of nutrients.

Results from Cochran *Q* and MR-Egger intercept tests demonstrated that there was no evidence of heterogeneity and horizontal pleiotropy in significant MR results. The MR-PRESSO analysis did not detect outlying SNPs. (See Table S4, Supplemental Digital Content, http://links.lww.com/MD/N578, which illustrates pleiotropy assessment for the results of obesity on circulating levels of nutrients.) The leave-one-out analyses displayed that the causal associations were not substantially influenced by any single SNP. (See Figure S2, Supplemental Digital Content, http://links.lww.com/MD/N577, which illustrates leave-one-out plots of significant and nominal significant estimates about obesity on circulating levels of nutrients.)

## 4. Discussion

Understanding the causal relationship between obesity and circulating levels of nutrients is of great significance for the prevention and health care of some diseases. To our knowledge, this is the first study evaluating the causal link between obesity and circulating levels of nutrients using 2-sample MR analysis. In the current MR analysis, we found genetically predicted higher BMI was associated with lower circulating levels of magnesium, folate, vitamin A, vitamin E, and albumin. On the other hand, there was also the evidence that genetically predicted higher WHR were associated with lower circulating levels of magnesium, folate, vitamin C, and albumin. The finding provides new evidence support for clinical nutritional prevention in patients with obesity.

In this study, our results indicated that obesity was related to an increased risk of magnesium deficiency. Magnesium deficiency is an easily overlooked electrolyte disorder. Magnesium deficiency can lead to a series of clinical symptoms, including depression, fatigue, muscle spasms, and arrhythmias, and increase the risk of developing osteoporosis and sarcopenia.^[26]^ Prior studies have indicated that magnesium deficiency is a highly prevalent in patients with obesity, type-2 diabetes, or metabolic syndrome.^[27]^ A prospective study of 5115 young adults with a 30-year longitudinal follow-up found magnesium intake is inversely associated with the incidence of obesity.^[28]^ Another study from the Mexican population also found magnesium intake is associated with lower BMI and waist circumference.^[29]^

Accumulation of evidence has shown that obesity leads to a decrease in the circulating levels of fat-soluble vitamins, such as vitamins A and E because they are stored in the adipose tissue.^[30]^ In this study, we also observed the causal effects of obesity on circulating levels of vitamins A and E. Similar to our results, Godala et al^[31]^ found that compared with normal-weight individuals, individuals with obesity had lower levels of plasma vitamin E. Inconsistently, a meta-analysis including 25 observational studies with 51,276 participants demonstrated that obesity was inversely associated with dietary vitamin E level, but not circulating vitamin E level.^[32]^ This may be due to bias caused by some confounding factors. Hence, our findings may offer help to clarify the relationship between obesity and circulating levels of vitamin E. In our study, we also found a causal association between obesity and circulating levels of folate and vitamin C. Folate deficiency can cause an increase in homocysteine concentration, thereby increasing the risk of cardiovascular disease.

Observational evidence has shown that obesity was associated with lower circulating levels of folate.^[33,34]^ More specifically, each unit increase in BMI causes a 1% decrease in serum folate concentration.^[35]^ Vitamin C is an essential antioxidant in the body. Previous studies have reported higher BMI led to lower C circulating levels, which corroborates our results.^[36,37]^

Low albumin levels are considered as an indicator of malnutrition, while obesity is usually considered as overnutrition. It’s hard to imagine obesity leading to a decrease in albumin levels. However, we observed that obesity was negatively associated with circulating levels of albumin. Several preliminary studies from bariatric surgery patients demonstrated that albumin deficiency was reported in 6.1% to 7.7%.^[38–40]^ Hypoalbuminemia is strongly associated with an increased risk of postoperative complications.^[41,42]^ However, another study showed hypoalbuminemia was related to nutritional status and age rather than BMI.^[43]^ Therefore, our findings could help to clarify this difference.

## 5. Strengths and limitations

The study first conducted MR analysis to comprehensively evaluate causal relationships between genetically predicted obesity and circulating levels of nutrients. The current study with data was restricted to populations of European ancestry, which reduces the bias of population stratification.

However, there are some limitations that need to be noted in this study. First, our MR study mainly analyzed individuals of European ancestry; hence, the findings may not precisely generalize to other ancestry. Previous epidemiological studies have also reported there were racial differences in nutritional deficiencies among patients with obesity.^[8]^ Second, we failed to further perform the sex-specific, age-specific, and BMI-specific analyses because of the lack of relevant GWAS summary databases. Third, due to the lack of relevant GWAS data, we are unable to analyze other related nutrients, such as vitamins B1, B2, B3, B5, vitamin K, sulfur, iodine, and sodium. Lastly, due to some inherent shortcomings in MR analysis, it is impossible to completely rule out horizontal pleiotropy.

## 6. Conclusions

In conclusion, our study provides evidence for causal associations between obesity and lower circulating levels of nutrients, including magnesium, folate, vitamin A, vitamin C, vitamin E, and albumin. Our findings highlight the necessity of adjuvant nutrients in obesity management.

## Acknowledgments

The authors are grateful to the authors of the original studies for sharing the genome-wide association studies (GWASs) summary statistics in the present study.

## Author contributions

**Conceptualization:** Wenhui Chen.

**Data curation:** Wenhui Chen, Guie Gao, Ruzhen Ou.

**Methodology:** Wenhui Chen, Guie Gao, Ruzhen Ou.

**Supervision:** Wenhui Chen.

**Writing—review & editing:** Wenhui Chen.

**Formal analysis:** Guie Gao.

**Writing—original draft:** Guie Gao, Ruzhen Ou.

**Investigation:** Ruzhen Ou.

**Resources:** Ruzhen Ou.

## Supplementary Material


