# Supplementary material for: Obesity influencing circulating levels of nutrients: Evidence from Mendelian randomization study
Source: Medicine (Baltimore). 2024 Sep 13;103(37):e39594. doi: 10.1097/MD.0000000000039594 (PMC11404870; doi:10.1097/MD.0000000000039594)
Supplement: Supplementary file 2 [file medi-103-e39594-s002.docx]

**Figure S1.** Scatter plots of significant and nominal significant estimates about obesity on circulating levels of nutrients. A BMI on Copper; B WHR on Calcium; C. BMI on Magnesium; D. WHR on Magnesium; E. WHR on Potassium; F. BMI on Folate; G. WHR on Folate; H. BMI on Vitamin A; I. WHR on Vitamin A; J. BMI on Vitamin C; K. WHR on Vitamin C; L. BMI on Vitamin E; M. WHR on Vitamin E; N. BMI on Vitamin B6; O. BMI on Albumin; P.WHR on Albumin

BMI: body mass index; WHR: Waist-to-hip ratio;

| A. BMI on Copper | B. WHR on Calcium |
| --- | --- |
| 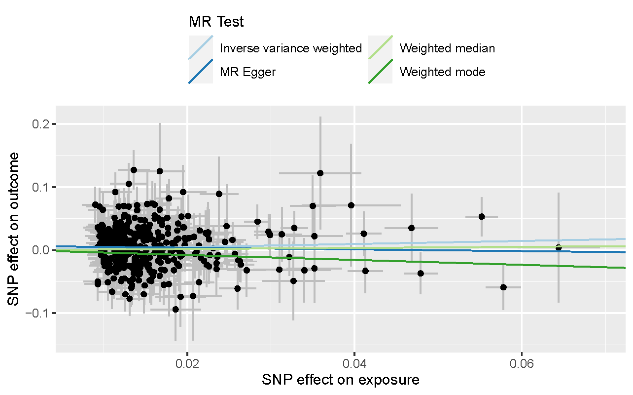 | 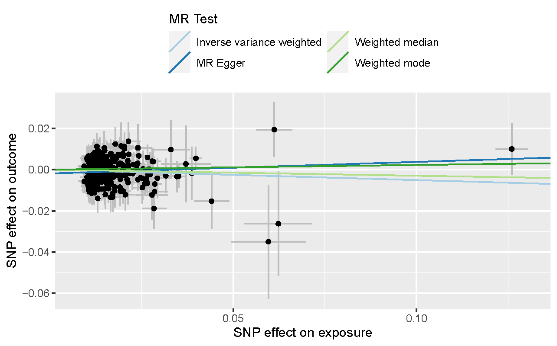 |
| C. BMI on Magnesium | D. WHR on Magnesium |
| 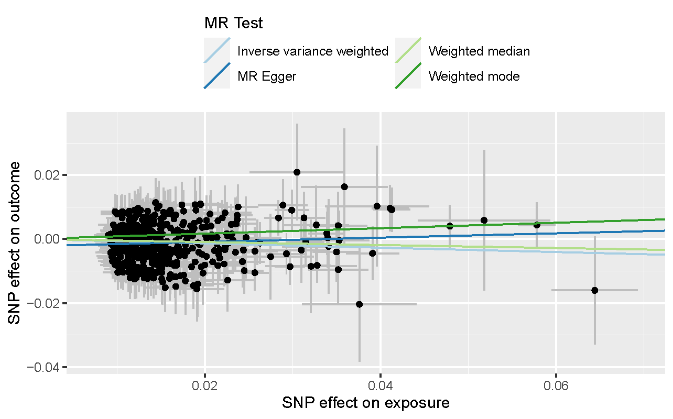 | 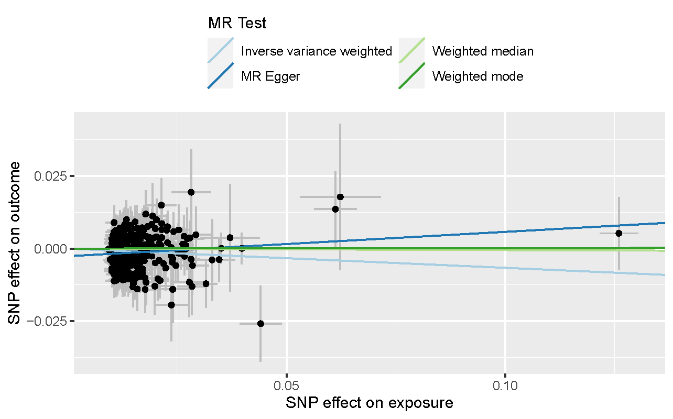 |
| E. WHR on Potassium | F. BMI on Folate |
| 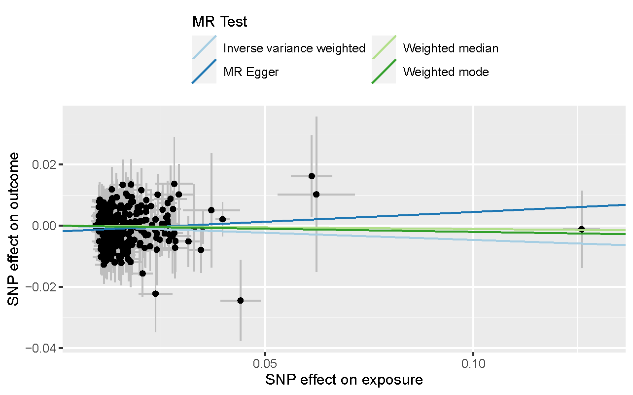 | 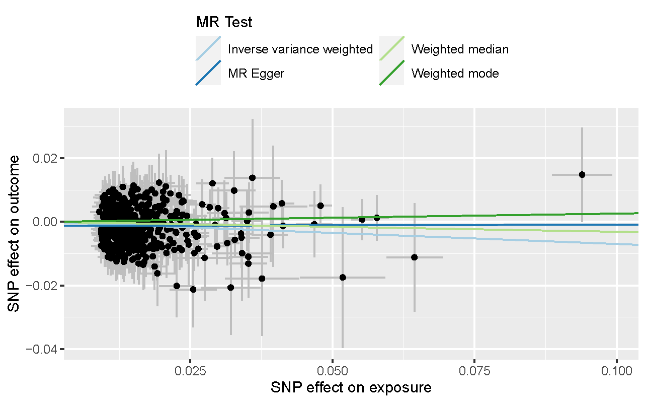 |
| G. WHR on Folate | H. BMI on Vitamin A |
| 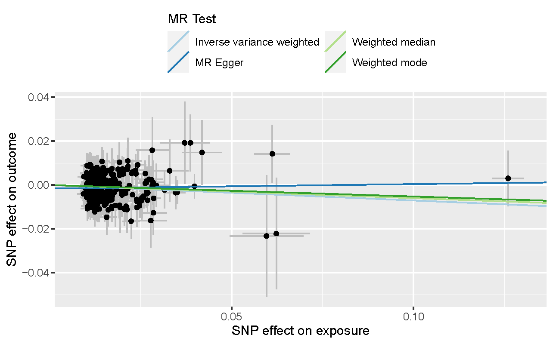 | 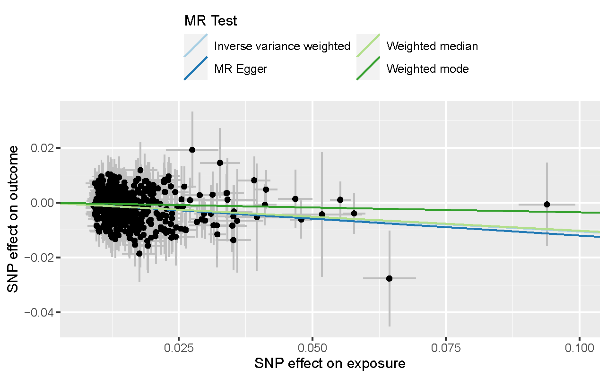 |
| I. WHR on Vitamin A | J. BMI on Vitamin C |
| 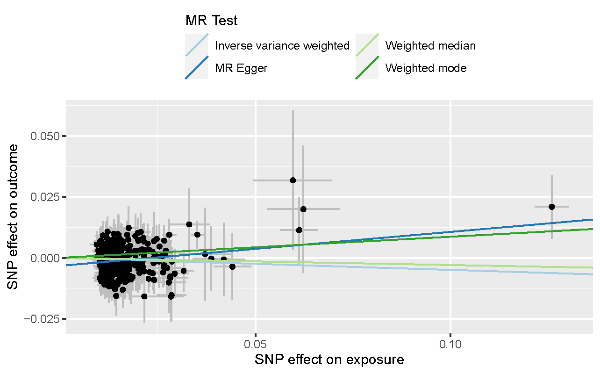 | 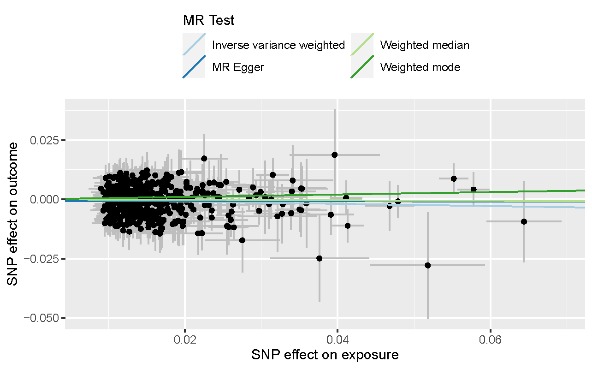 |
| K. WHR on Vitamin C | L. BMI on Vitamin E |
| 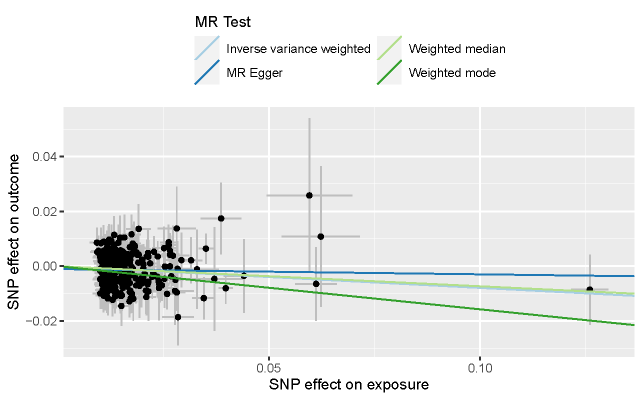 | 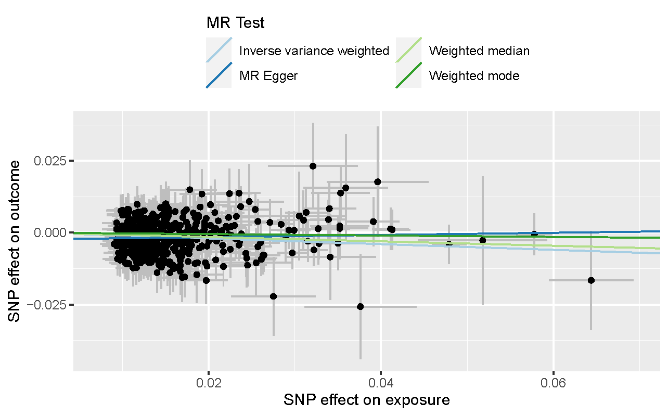 |
| M. WHR on Vitamin E | N. BMI on Vitamin B6 |
| 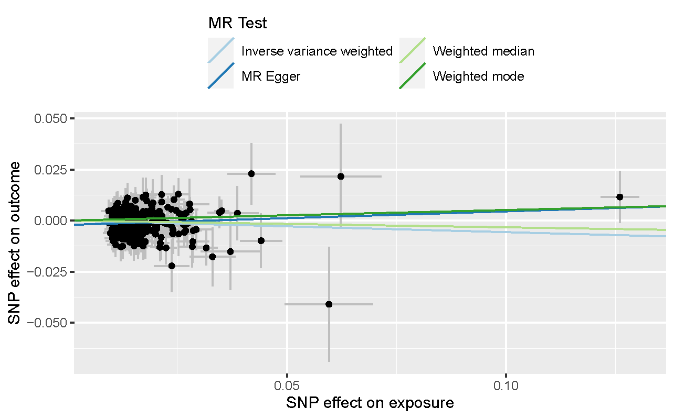 | 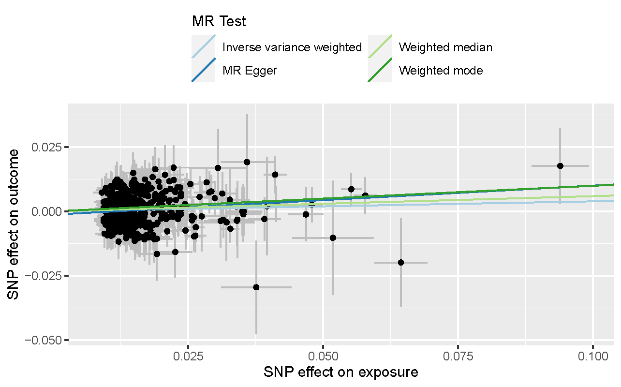 |
| O. BMI on Albumin | P.WHR on Albumin |
| 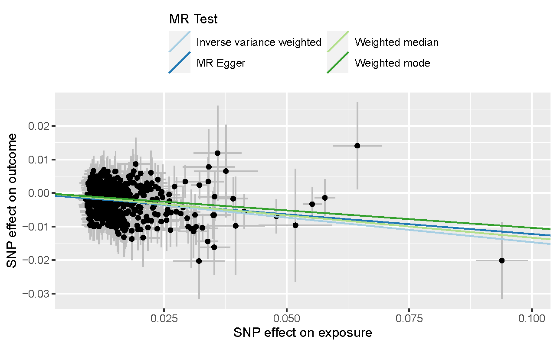 | 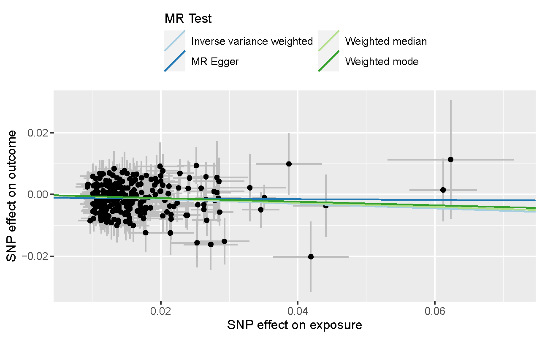 |

**Figure S2.** Leave-one-out plots of significant and nominal significant estimates about obesity on circulating levels of nutrients. A BMI on Copper; B WHR on Calcium; C. BMI on Magnesium; D. WHR on Magnesium; E. WHR on Potassium; F. BMI on Folate; G. WHR on Folate; H. BMI on Vitamin A; I. WHR on Vitamin A; J. BMI on Vitamin C; K. WHR on Vitamin C; L. BMI on Vitamin E; M. WHR on Vitamin E; N. BMI on Vitamin B6; O. BMI on Albumin; P.WHR on Albumin

BMI: body mass index; WHR: Waist-to-hip ratio;

| A. BMI on Copper | B. WHR on Calcium |
| --- | --- |
| 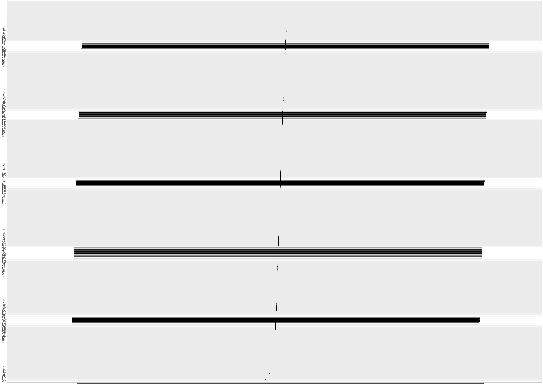 | 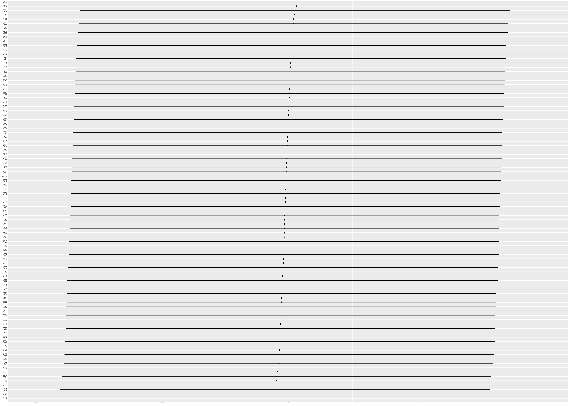 |
| C. BMI on Magnesium | D. WHR on Magnesium |
| 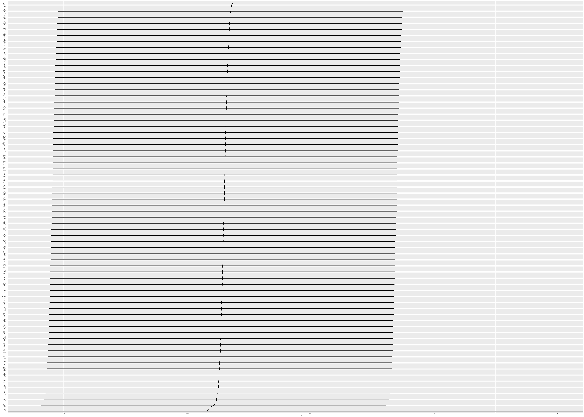 | 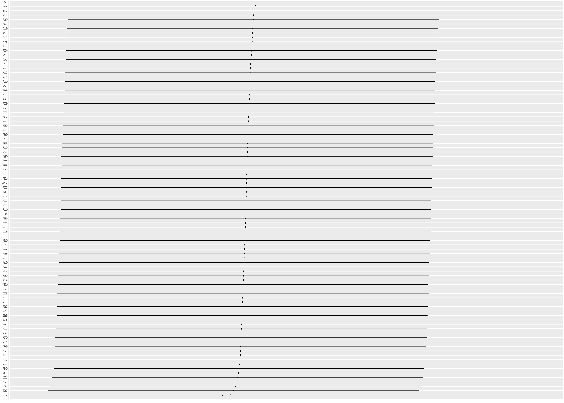 |
| E. WHR on Potassium | F. BMI on Folate |
| 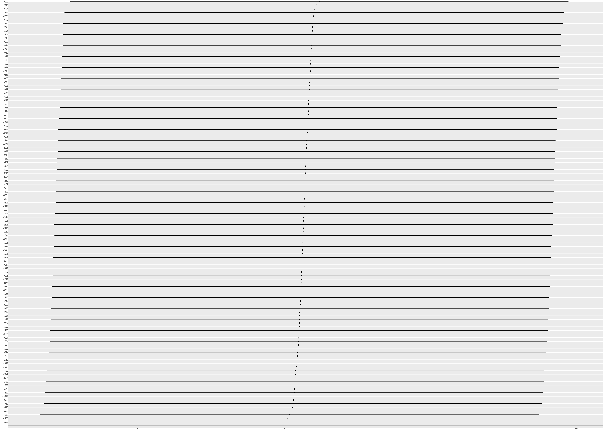 | 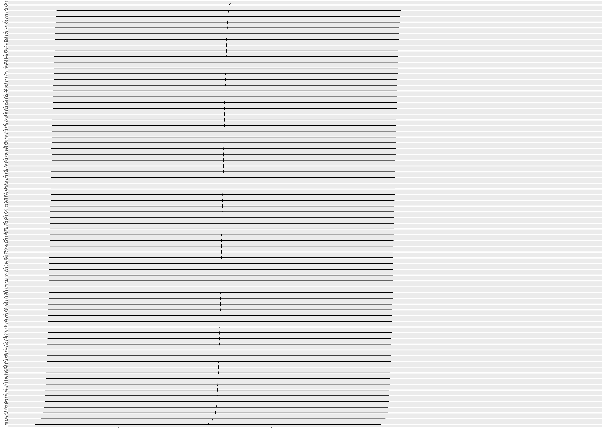 |
| G. WHR on Folate | H. BMI on Vitamin A |
| 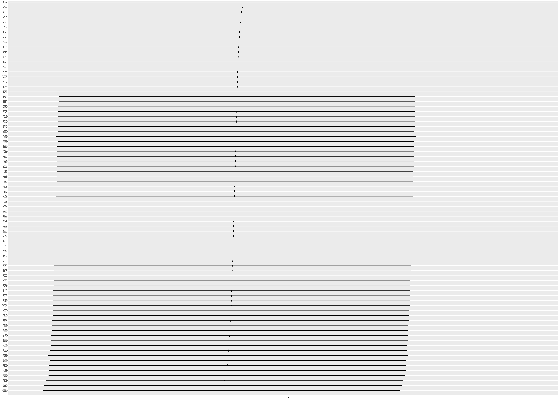 | 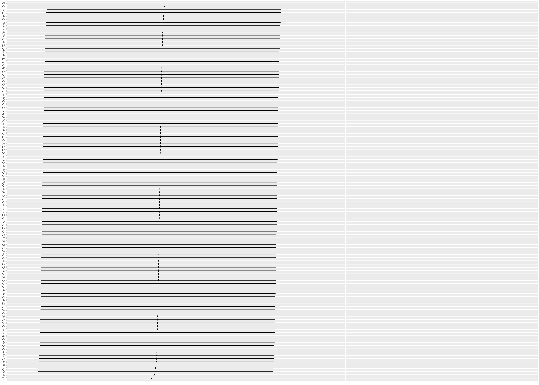 |
| I. WHR on Vitamin A | J. BMI on Vitamin C |
| 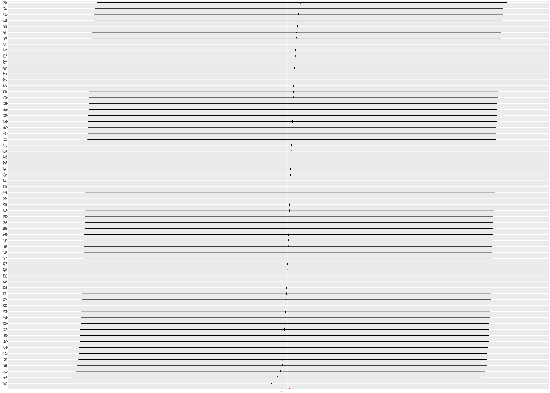 | 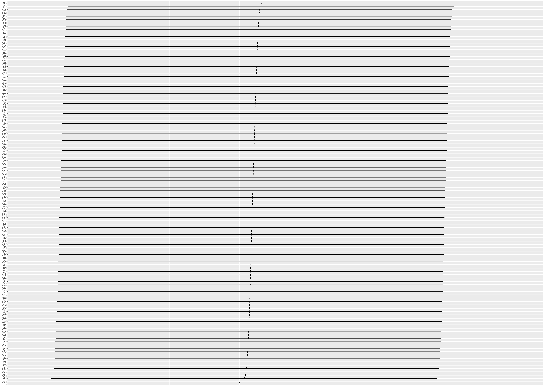 |
| K. WHR on Vitamin C | L. BMI on Vitamin E |
| 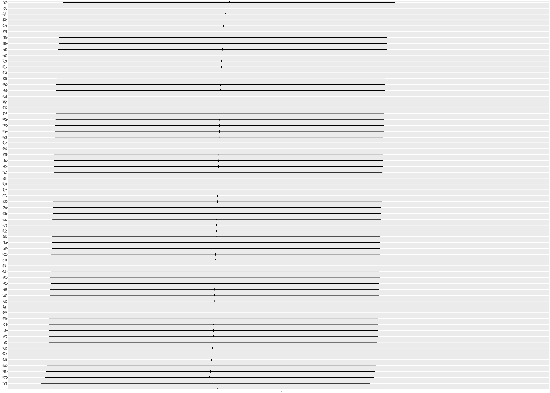 | 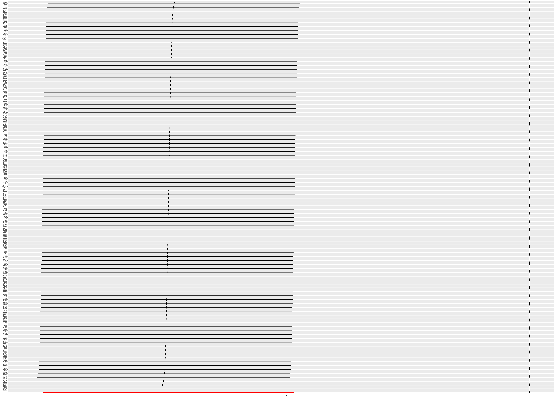 |
| M. WHR on Vitamin E | L. BMI on Vitamin B6 |
| 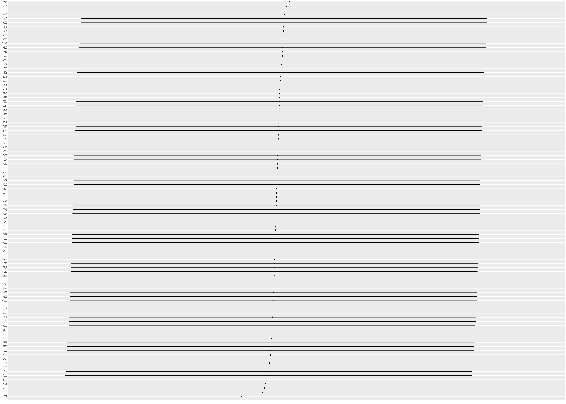 | 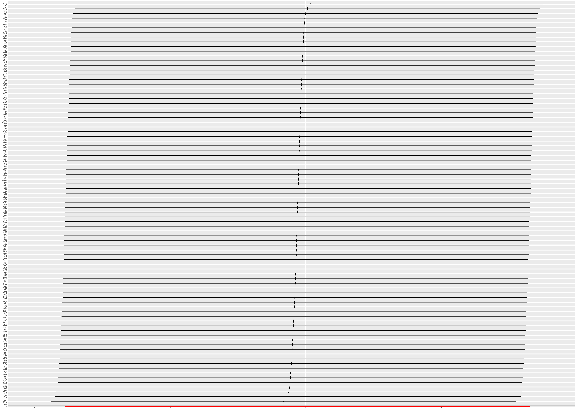 |
| O. BMI on Albumin | P.WHR on Albumin |
| 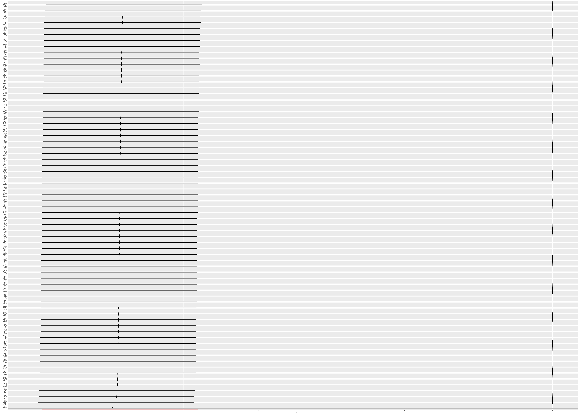 | 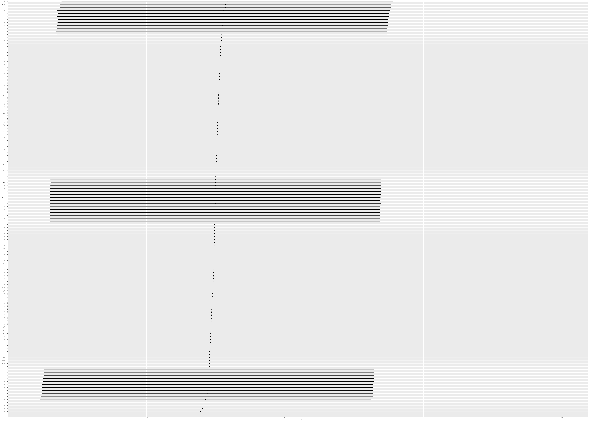 |
